# Thyroid hormone receptor- and stage-dependent transcriptome changes affect the initial period of *Xenopus tropicalis* tail regeneration

**DOI:** 10.1186/s12864-024-11175-4

**Published:** 2024-12-31

**Authors:** Shouhong Wang, Liezhen Fu, Bin Wang, Yanmei Cai, Jianping Jiang, Yun-Bo Shi

**Affiliations:** 1 Chengdu Institute of Biology, Chinese Academy of Sciences, Chengdu, 610041 China; 2 Section On Molecular Morphogenesis, Eunice Kennedy Shriver National Institute of Child Health and Human Development, National Institutes of Health, Bethesda, MD 20892 USA; 3 College of Life Science, Sichuan Normal University, Chengdu, 610101 China

**Keywords:** Thyroid hormone, TR knockout, Regeneration, Tail, Gene regulation, *Xenopus tropicalis*

## Abstract

**Background:**

Thyroid hormone (T3) has an inhibitory effect on tissue/organ regeneration. It is still elusive how T3 regulates this process. It is well established that the developmental effects of T3 are primarily mediated through transcriptional regulation by thyroid hormone receptors (TRs). Here we have taken advantage of mutant tadpoles lacking both TRα and TRβ (TRDKO), the only receptor genes in vertebrates, for RNA-seq analyses to investigate the transcriptome changes underlying the initiation of tail regeneration, i.e., wound healing and blastema formation, because this crucial initial step determines the extent of the functional regeneration in the later phase of tissue regrowth.

**Results:**

We discovered that GO (gene ontology) terms related to inflammatory response, metabolic process, cell apoptosis, and epithelial cell migration were highly enriched among commonly regulated genes during wound healing at either stage 56 or 61 or with either wild type (WT) or TRDKO tadpoles, consistent with the morphological changes associated with wound healing occurring in both regenerative (WT stage 56, TRDKO stage 56, TRDKO stage 61) and nonregenerative (WT stage 61) animals. Interestingly, ECM-receptor interaction and cytokine-cytokine receptor interaction, which are essential for blastema formation and regeneration, were significantly enriched among regulated genes in the 3 regenerative groups but not the non-regenerative group at the blastema formation period. In addition, the regulated genes specific to the nonregenerative group were highly enriched with genes involved in cellular senescence. Finally, T3 treatment at stage 56, while not inducing any measurable tail resorption, inhibited tail regeneration in the wild type but not TRDKO tadpoles.

**Conclusions:**

Our study suggests that TR-mediated, T3-induced gene regulation changed the permissive environment during the initial period of regeneration and affected the subsequent patterning/outgrowth period of the regeneration process. Specifically, T3 signaling via TRs inhibits the expression of ECM-related genes while promoting the expression of inflammation-related genes during the blastema formation period. Interestingly, our findings indicate that amputation-induced changes in DNA replication-related pathways can occur during this nonregenerative period. Further studies, particularly on the regenerative microenvironment that may depend on ECM-receptor interaction and cytokine-cytokine receptor interaction, should provide important insights on the regulation of regenerative capacity during vertebrate development.

**Supplementary Information:**

The online version contains supplementary material available at 10.1186/s12864-024-11175-4.

## Background

Adult mammals face a challenge in their recovering from tissue and organ injuries, primary due to their extremely limited regenerative capabilities. Only a few tissues and organs, such as the liver, distal digits and antlers, possess the ability to regenerate after damage [1–4]. Conversely, lower vertebrates, including teleost fish and amphibians, exhibit remarkable regenerative abilities across multiple organs, such as tail, heart, brain and limb [5–9]. These organisms, with robust regenerative capabilities, provide a valuable opportunity to investigate the mechanisms underlying the regenerative failure in higher vertebrates, ultimately paving the way for the development of regenerative therapies for human applications.

Current research on regeneration involves developing a wide spectrum of model organisms to decipher factors and mechanisms involved in tissue repair and scarring [7, 10]. However, most studies on tissue and organ regeneration are mainly focused on lower vertebrates, such as zebrafish and axolotl, due to their life-long remarkable capacity to heal in a scar-free manner or on mammals with limited regenerative capabilities [11–14]. Anurans such as *Xenopus tropicalis* (*X. tropicalis*), occupy an intermediate phylogenetic position between lower vertebrates and higher vertebrates, serving as a crucial bridge in understanding the evolutionary shift in regenerative capabilities between fish/urodeles and mammals. Importantly, *Xenopus* has high regenerative capacity at the larval/premetamorphic stages, but this capacity is largely lost during metamorphosis, thus offering a unique opportunity to investigate the mechanisms underlying the switch from regenerative to non-regenerative organs within the lifespan of a single organism [5, 15].

*Xenopus* metamorphosis is controlled by thyroid hormone (T3) via thyroid hormone receptor (TR)-mediated transcriptional regulation of target genes [16–21]. Previous studies have revealed a negative effect of T3 on the regeneration of body appendages in several animal models such as *Xenopus*, salamander and zebrafish [7, 22, 23]. Furthermore, our research has implicated the TR-mediated, T3-induced gene regulation program in the loss of tail regeneration ability, particularly during the patterning or regrowth phases in *X. tropicalis* [5]. However, it is still unclear how T3 via TRs affects the transcriptomic landscape important for the initiation of regeneration, which plays an essential role in governing tissue regrowth and functional recovery.

Here, we used the stage-matched wild-type (WT) and TR-double knockout (TRDKO), lacking both TRα and TRβ, animals at premetamorphic (stage 56) and metamorphic climax (stage 61) for RNA-seq analyses to investigate the transcriptome changes underlying the initiation of tail regeneration, i.e., wound healing (0–6 h post amputation) and blastema formation (6–24 h post amputation). These stages were chosen because we have previously shown that WT tadpoles at stage 56 (WT56) and TRDKO56) and TRDKO61 can regenerate the tail after amputation while WT61 tadpoles cannot, even though the formation wound epidermis for wound healing and the presence of a small tail bud with a cluster of proliferating cells for blastema formation at both stages in both wild type and TRDKO animals [5]. Consistent with earlier findings that the inflammation response and ECM-remodeling are known to be essential for blastema and regeneration, we discovered that genes involved in cytokine-cytokine receptor interaction and ECM-receptor interaction were significantly enriched among genes regulated during blastema period of regeneration in the three regenerative groups (WT56, TRDKO56, TRDKO61) but not in the non-regenerative group (WT61). In addition, the regeneration-regulated genes specific to the nonregenerative group were highly enriched with genes involved in cellular senescence. Our findings suggest that T3, via TR, regulates a gene expression program to induce cellular senescence, which may help to alter the permissive environment during the blastema formation period and thus inhibit subsequent patterning/outgrowth during the regeneration process.

## Materials and methods

### Experimental animals

Adults *X. tropicalis* of wild type were purchased from NASCO (Fort Atkinson, WI, USA; http://www.enasco.com) and subsequently reared in our laboratory. Tadpole developmental stages were determined according to [24]. To generate tadpoles with a double knockout of TRα and TRβ (TRDKO (TRα (-/-)β(-/-)), sexually mature *X. tropicalis* frogs that were homozygous for the TRα knockout and heterozygous for the TRβ knockout (TRα (-/-)β( ±)) were mated. The tadpoles at stage 56 were treated with 5 nM T3 for 0 (control) or 3 days and maintained at 25 °C. All animal care and treatments were performed as approved by the Animal Use and Care Committee of *Eunice Kennedy Shriver* National Institute of Child Health and Human Development of the National Institutes of Health.

### Genotyping

Tadpoles were anesthetized using a 0.02% solution of MS222 (TCI, Tokyo, Japan). Next, after anesthesia, a small tail tip (about 5 mm or less) was clipped from the tadpole and lysed in 20 μl of QuickExtract DNA extraction solution (EPICENTRE Biotechnologies, Madison, WI, USA) at a temperature of 65 °C for 20 min (min). Following an incubation at 95 °C for 2 min and subsequent centrifugation at 4000 rpm for 10 min. 1 μl of the extracted DNA solution was then utilized for genotyping [19]. For genotyping, a PCR-based approach was employed to distinguish between the TRβ wild type and a mutant variant with a 19-base deletion with the forward primer, 5´-GGACAACATTAGATCTTTCTTTCTTTG-3´ and reverse primer, 5´-CACACCACGCATAGCTCATC-3´, respectively. The PCR cycling conditions were 33 cycles of 94 °C for 30 s, 60 °C for 30 s, and 72 °C for 20 s. The PCR products were then analyzed using electrophoresis on a 3.5% agarose gel, allowing the determination of the genotype based on the sizes differences of the PCR products.

### Amputation procedure

Tadpoles at the indicated stages were first anesthetized in a solution of 0.02% MS222 diluted in 0.1 MMR (0.1 M NaCl, 2.0 mM KCl, 1 mM MgSO4, 2 mM CaCl2, 5 mM HEPES (pH 7.8). Briefly, after anesthesia and following the previously procedures [5], a sterile scalpel was used to remove around 30–50% of the tadpole tail, whichever was less, at both stages 56 and 61. The amputated tadpoles were placed in a tank with rearing water and maintained in 25℃ incubators.

### RNA-seq analysis

At 0 h, 6 h and 24 h post-amputation of the tail of wild type and TRDKO tadpoles at stage 56 and stage 61, the part of the tail including all regenerated tail plus about 250 μm of the original uncut tail proximal to the site of amputation was dissected for total RNA isolation with the Direct-zol RNA MiniPrep kit (Zymo research, Catalog No: ZR2052). For RNA-seq analysis, wild type tadpoles included stage 56 amputated tail at 0 h (*n* = 9), 6 h (*n* = 6) and 24 h (*n* = 6) and stage 61 amputated tail at 0 h (*n* = 5), 6 h (*n* = 4) and 24 h (*n* = 5); and TRDKO tadpoles used included stage 56 amputated tail at 0 h (*n* = 5), 6 h (*n* = 6) and 24 h (*n* = 5) and stage 61 amputated tail at 0 h (*n* = 5), 6 h (*n* = 4) and 24 h (*n* = 5). The RNA samples were submitted to NICHD Molecular Genomics Core to prepare two biological replicates for each RNA sample in library construction of poly-A-selected RNA with the TruSeq Stranded Total RNA Library Prep Kit (Illumina Inc., San Diego, CA, USA). Briefly, the mRNA was isolated from total RNA by using poly-T oligo-attached magnetic beads and chemically fragmented. First-strand cDNA was synthesized by using random hexamer primers and M-MLV Reverse Transcriptase (RNase H-). Second-strand cDNA synthesis was subsequently performed by using DNA Polymerase I and RNase H. The libraries were sequenced on the Illumina NovaSeq 6000 platform to obtain 2 × 100 bp paired-end reads. The demultiplexed and adapter-removed short reads were mapped to NCBI Xenopus tropicalis genome assembly, Xenbase *Xenopus tropicalis* Genome assembly (v10.0), and Ensembl *Xenopus tropicalis* Genome (JGI 4.2) with STAR software (version 2.6.1c) and read counts for each gene/exon were obtained with featureCounts tool of Subread software (version 1.6.3). The raw fastq data were deposited to the NCBI GEO database with accession number GSE225045.

### Bioinformatics analysis

R Bioconductor DESeq2 package was used for differential gene expression (DEGs) analysis of the count data above. The relative expression value of DEGs in each sample was normalized to fragments per kilobase of the transcript per million fragments mapped (FPKM). Venny 2.1 (https://bioinfogp.cnb.csic.es/tools/venny/) was used for visualizing the overlapped genes. The heatmap of up-and down-regulated genes are analyzed by VolcaNoseR (https://huygens.science.uva.nl/VolcaNoseR/). Gene Ontology (GO) analysis and KEGG pathway enrichment analysis of the DEGs were performed with MetaCore software and KOBAS 3.0 bioinformatics resource (http://bioinfo.org/kobas/genelist/), respectively.

### Quantitative reverse-transcription PCR (RT-qPCR)

Total RNA was extracted from the tails of wild type and TRDKO tadpoles at stage 56 and stage 61 at 0 h, 6 h and 24 h post amputation, using the Direct-zol RNA MiniPrep kit (Zymo research). Next, 200–500 ng of total RNA was reverse transcribed into cDNA using a High-Capacity cDNA Reverse Transcription kit (Applied Biosystems, Waltham, MA). Expression levels were quantified using the ΔΔCt method, with *rpl8* used as the reference gene [25]. The expression analyses were performed at least twice, with consistent results. The primer sequences used are listed in Supplemental Table 1 (Table S1).

### Statistical analysis

The data are presented as mean ± standard error (SE). Statistical significance between groups was assessed using Student’s t-test, performed with Prism 9 software (GraphPad Software, La Jolla, CA, USA).

## Results

### Transcriptome analysis reveals conserved gene regulation program and biological processes during wound healing in* Xenopus tropicalis* tail

**Fig. 1:**
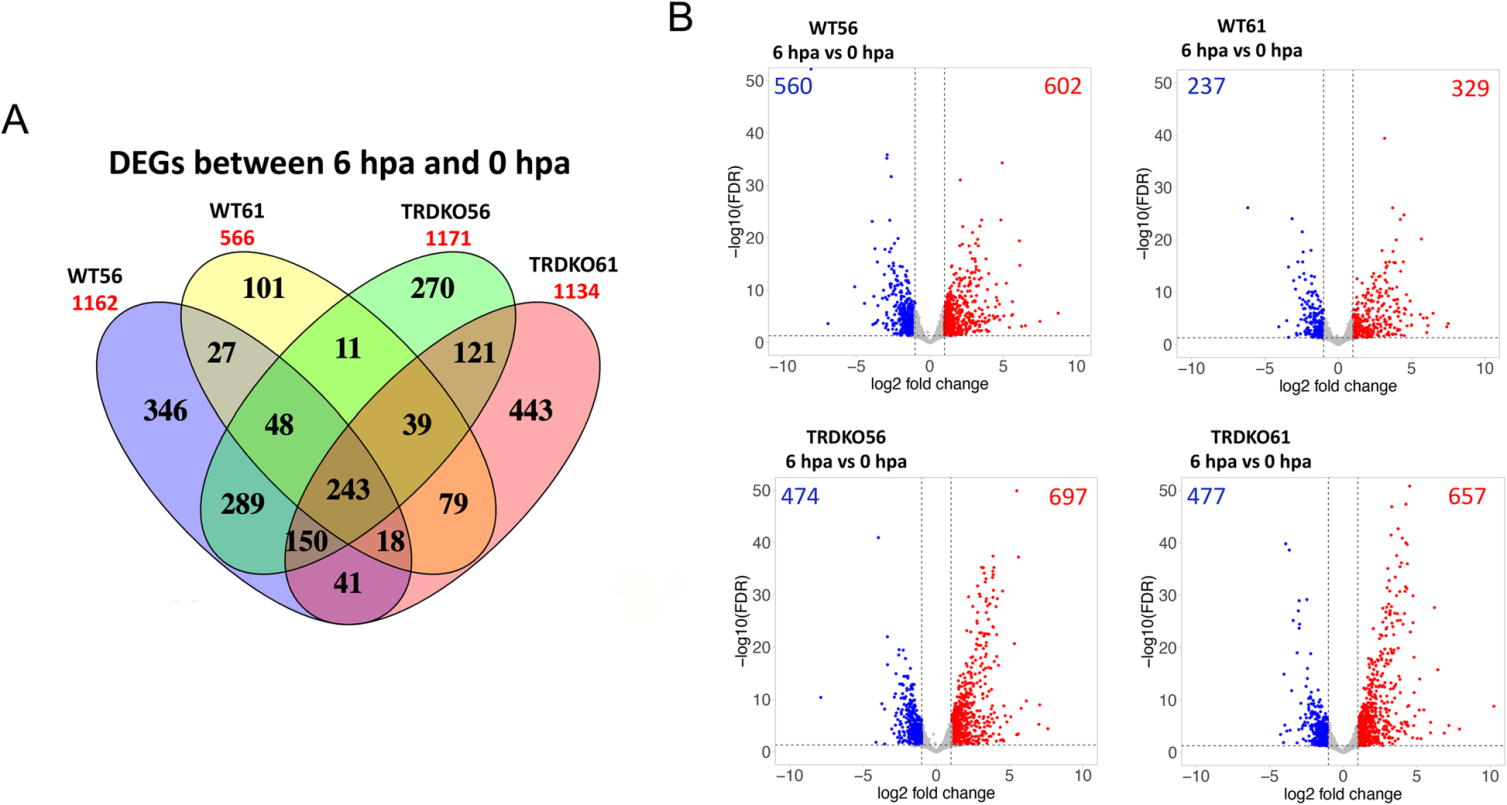
Fewer differentially expressed genes (DEG) between 0 and 6 h postamputation (hpa) (the wound healing period) in the tail of wild-type tadpoles at the climax (stage 61) than in the other 3 groups of animals (wild-type at stage 56, TRDKO at stage 56 and TRDKO at stage 61). **A** Venn diagram of the DEGs identified in the wild-type and TRDKO tail at stage 56 and stage 61 during wound healing period. Note that there were 566 DEGs at wild-type stage 61 compared to 1162 DEGs at wild-type stage 56, 1171 DEGs at TRDKO stage 56 and 1134 DEGs at TRDKO stage 61. **B** Volcano plots showing the up (red)- and down (blue)-regulated DEGs in the wild-type and TRDKO tail at premetamorphosis (stage 56) and metamorphic climax (stage 61) during wound healing

We have previously shown that tadpoles of wild type at premetamorphic stage 56 (WT56), TRDKO56, and TRDKO61 were regenerative while those of WT61 were nonregenerative after tail amputation [5], indicating that TR is critical for the loss of tail regeneration capacity at the metamorphic climax. To investigate the underlying molecular mechanism, we focused on the gene expression program during the initial period of *Xenopus* tail regeneration, i.e., wound healing and blastema formation (0–6 and 6–24 h post amputation, respectively). We carried out RNA-seq analysis on the regenerated tail, including all regenerated tissues plus 250 $\mu m$ of the original uncut tail proximal to the site of amputation, from wild-type and TRDKO tadpoles at both stage 56 and stage 61. We detected 26,531 genes (Table S2) and identified 1162 (602 upregulated, 560 downregulated), 1171 (697 upregulated, 474 downregulated), and 1134 (657 upregulated, 477 downregulated) differentially expressed genes (DEGs, log2 FC > 1, FDR < 0.05) in regenerative animals of WT56, TRDKO56, TRDKO61, respectively, but only 566 (329 upregulated, 237 downregulated) DEGs in nonregenerative animals (WT61) during wound healing (6 h post amputation (hpa) vs 0 hpa) (Fig. 1 and Table S3A-D).

We next investigated four genes, *leptin*, *il11*, *mmp1* and *sox9*, known to be regulated during wound healing, and found that 3 (*leptin*, *il11*, *mmp1)* were upregulated and *sox9* was downregulated by 6 h after amputation in (Fig. S1). With the exception of the lack of regulation of *sox9* in the non-regenerative WT61 group, the data were entirely consistent with earlier findings in *Xenopus* or other organisms [5, 26–28].

**Fig. 2:**
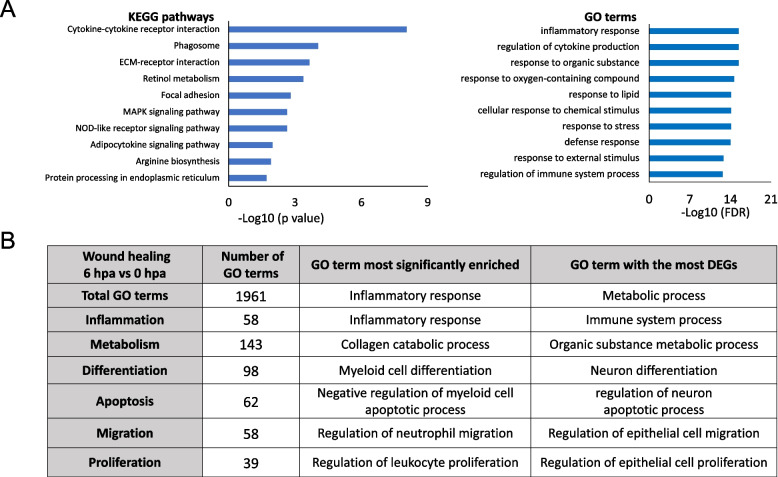
KEGG and GO analyses reveal that conserved KEGG pathways/GO terms are regulated in wild-type and TRDKO tadpoles during wound healing. **A** Top 10 KEGG pathways/GO terms significantly enriched among the 243 DEGs common to all 4 groups (wild-type stage 56, wild-type stage 61, TRDKO stage 56 and TRDKO stage 61) during wound healing (6 hpa vs 0 hpa). Notably, the inflammatory response is the most significantly enriched pathways/terms. **B** The GO terms known to be involved in wounding healing were highly enriched among 243 DEGs common to all 4 groups

As we observed that both regenerative (WT56, TRDKO56, TRDKO61) and nonregenerative (WT61) animals could complete the wound healing [5], we analyzed the 243 DEGs common among the four groups of regenerating tails during the wound healing period (Fig. 1, Table S3E). We found that the most significantly enriched KEGG pathway and GO term among these DEGs were “cytokine-cytokine interaction” and “inflammatory response”, respectively, which are known to be involved in wound healing (Fig. 2A, Table S4). Furthermore, six broad categories of GO terms most relevant to wound healing, i.e., inflammation, metabolism, differentiation, apoptosis, migration and proliferation [29–33], were all highly enriched among the totally 1961 enriched GO terms (Fig. 2B, Table S4B). Each of the categories had many GO terms enriched among the DEGs (Fig. 2B, Table S4B). Our results demonstrated conservations in the involvement processes during wound healing in *Xenopus*. They further suggest that TR is not involved in regulating wound healing during *Xenopus* tail regeneration.

### Distinct transcriptional regulation programs exist in the regenerative and nonregenerative tail during blastema formation period

**Fig. 3:**
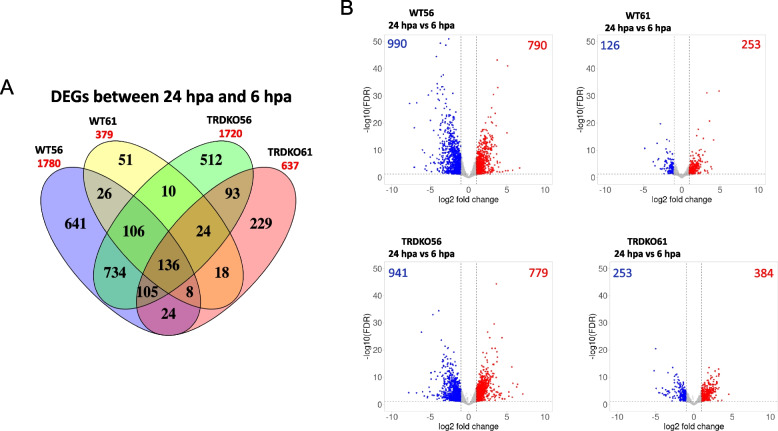
More DEGs during the blastema period after tail amputation at premetamorphic stages than those at the metamorphic climax for both wild-type and TRDKO animals. **A** Venn diagram of the DEGs identified in the wild-type and TRDKO tail at stage 56 and stage 61 during the blastema period (between 6 and 24 hpa). Note that there were 379 DEGs at wild-type stage 61 and 637 DEGs at TRDKO stage 61 tail compared to 1780 DEGs and 1720 DEGs at wild-type stage 56 and TRDKO stage 56, respectively. **B** Volcano plot showing the up (red)- and down (blue)-regulated DEGs in the wild-type and TRDKO tail at premetamorphosis (stage 56) and metamorphic climax (stage 61) during blastema period

Various studies have established that blastema formation is a critical step in bridging the wound epidermis and tissue outgrowth [34, 35]. Thus, we carried transcriptome analysis and identified 1780 (790 upregulated and 990 downregulated), 1720 (779 upregulated and 941 downregulated) and 637 (384 upregulated and 253 downregulated) DEGs in regenerative animals WT56, TRDKO56, TRDKO61, respectively, but only 379 (253 upregulated and 126 downregulated) DEGs in nonregenerative animals (WT61) during blastema formation (24 hpa vs 6 hpa) (Fig. 3 and Table S5). Among them, two categories of DEGs are of particular interest. The first one is the 136 DEGs commonly regulated in four groups and the other one is the 105 DEGs only regulated in the regenerative animals (Fig. S2A). KEGG pathway and GO analyses on the 136 and 105 DEGs, respectively, revealed that DNA replication and cell cycle were the most significantly enriched among the 136 common DEGs (Fig. S2B). On the other hand, cytokine-cytokine receptor interaction and ECM-receptor interaction pathways and GO terms, in addition to those related to DNA replication and cell cycle, were highly enriched among the 105 DEGs specific to the regenerative groups (Fig. S2C). These results indicate TR-mediated, T3-induced the regulated DEGs involved in regulating the inflammatory response and ECM remodeling are critical for blastema formation.

### Analyses of stage-matched wild-type and TRDKO animals at metamorphic climax reveal the TR-mediated T3-induced gene expression program regulating blastema formation

**Fig. 4:**
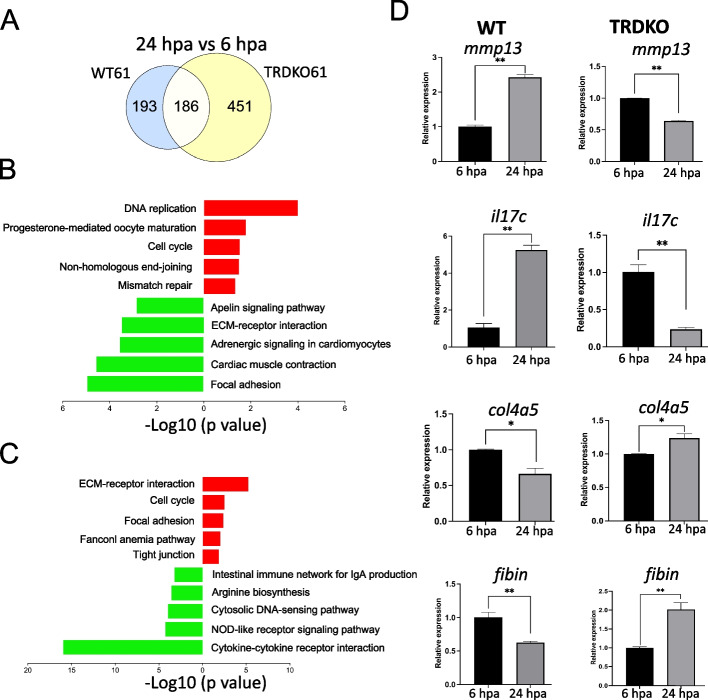
The expression of genes related to ECM-receptor interaction pathway is increased and that of genes involved in cytokine-cytokine receptor interaction pathway is reduced during the blastema period of regeneration in TRDKO tail at stage 61, the opposite to that in wild-type tail at stage 61. **A** Venn diagram of the DEGs in wild-type and TRDKO tail at stage 61 during the blastema period of regeneration. Note that there were more DEGs in the tail of TRDKO tadpoles than wild-type (WT) tadpoles. **B**/**C** Top 10 KEGG pathways significantly enriched among the 81 upregulated (pathways in red) or 112 downregulated (pathways in green) genes specific to WT tail (B) and the 219 upregulated (pathways in red) and 232 downregulated (pathways in green) genes specific to TRDKO tail (**C**). **D** Validation of the known inflammatory genes (*mmp13*, *il17c*) and ECM genes (*col4a5, fibin*) by RT-qPCR, normalized to that of *rpl8*

To further investigate if TR-mediated, T3-induced gene expression program is responsible for the inhibition of blastema formation, we focused on stage-matched WT61 and TRDKO61 animals, thereby eliminating any potential stage-dependent effects. We first identified 193 and 451 DEGs specifically regulated in WT61 and TRDKO61 tail, respectively, during blastema formation (between 6 and 24 hpa) (Fig. 4A). Next, KEGG pathway analyses were carried out separately on the upregulated 81 and downregulated 112 genes among the 193 DEGs of WT61 (Fig. 4B, Table S6), or the 219 upregulated genes and 232 downregulated genes among the 451 DEGs of TRDKO61 (Fig. 4C, Table S6) during blastema formation. The results showed that with the exception of cell cycle commonly enriched among upregulated genes in both genotypes, the rest of the top 5 most significantly enriched pathways were totally different between WT and TRDKO tails. Of interest, the ECM-receptor interaction pathway, important for regeneration, was significantly enriched among downregulated genes in the WT tail but highly enriched among the upregulated genes in TRDKO tail (Fig. 4 B-C, Fig.S3, Table S6). On the other hand, cytokine-cytokine receptor interaction was highly enriched among the downregulated genes in TRDKO tail but not in WT tail (Fig. 4B-C, Table S6), consistent with the need to down-regulate proinflammatory genes during the blastema formation period. In addition, RT-qPCR analysis showed that the expression of 2 proinflammatory genes, *mmp14* and *il17c*, and 2 ECM remodeling genes, *col4a5* and *fibin* in the regenerated portion of the tail was significantly upregulated and downregulated, respectively, between 24 and 6 h after amputation in wild type tail. In contrast, in the TRDKO tail, an opposite gene regulation pattern was observed (Fig. 4D), confirming the transcriptome data (Table S2. B, D) and demonstrating a critical role of TR in regulating gene expression during regeneration at the climax of metamorphosis.

### TR-mediated, T3-induced cellular senescence might be responsible for the inhibition of tail regeneration at the climax of metamorphosis

**Fig. 5:**
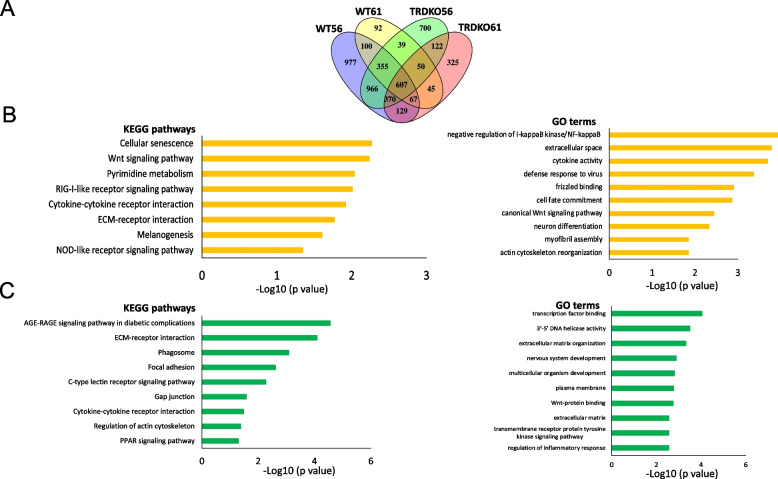
KEGG and GO analyses reveal common and distinct KEGG pathways/GO terms regulated during the initial period of tail regeneration. **A** Venn diagram of all DEGs identified by pair-wise comparisons among 0 hpa, 6 hpa and 24 hpa tail samples of 4 groups after amputation. **B** 8 KEGG pathways and top 10 GO terms significantly enriched among the 92 DEGs specific to WT st61 (stage 61) tail. Note that the most significantly enriched KEGG pathway is cellular senescence. **C** 9 KEGG pathways and top 10 GO terms significantly enriched among the 370 DEGs common to WT st56, TRDKO st56 and TRDKO st61 tail. Note that development related pathways are significantly enriched

To understand the global gene regulation and involved biological processes during the initial period of tail regeneration, i.e., wound healing and blastema formation, we combined the DEGs during wound healing and blastema formation for the different animal groups. Overall, there were 2941, 3209, 1715 and 1355 DEGs for WT56, TRDKO56, TRDKO61, and WT61 groups, respectively. Among them, 607 DEGs were common among the four group animals while 370 and 92 DEGs were unique to regenerative animals (WT56, TRDKO56, TRDKO61) and nonregenerative animals (WT61) (Fig. 5A), respectively.

**Fig. 6:**
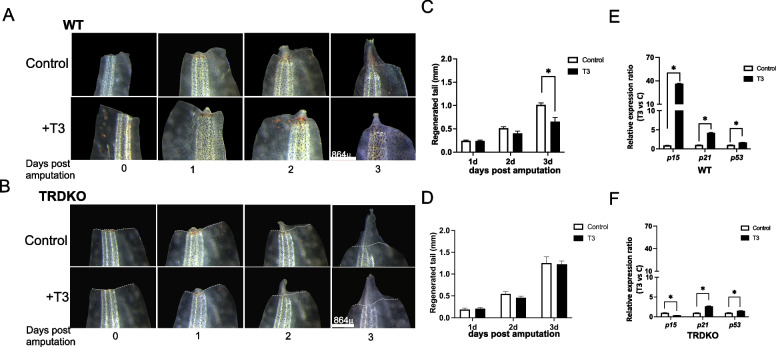
T3 treatment inhibits tail regeneration in wild-type tadpoles but not in TRDKO tadpoles at stage 56. **A**/**B** Morphological observations during tail regeneration in wild-type (A) and TRDKO (B) tadpoles showed that T3 treatment at stage 56 inhibited the regeneration compared to -T3 control, particularly at 3 days after tail amputation of wild-type while not TRDKO animals. The white dashed lines indicated amputation sites. Scale bar: 864 μm. **C**/**D** Quantitative analyses of the length of the regenerated tail in the wild-type (C) and TRDKO (D) tadpoles revealed that T3 treatment significantly decreased the regenerated length at 3 days post amputation in wild-type but not TRDKO animals. The data were shown as mean values of at least 3 replicates with SE. *P < 0.05. **E**/**F** Differential regulation of cellular senescence marker genes, *p15, p21* and *p53,* by T3 in WT56 and TRDKO56 animals during regeneration. Total RNA was isolated from regenerating tail of WT56 (E) and TRDKO56 (F) animals in the presence or absence of T3 for 3 days and analyzed by RT-qPCR for the expression of *p15, p21* and *p53*. The expression level was normalized to that of *rpl8,* with the expression in the absence of T3 set to 1 for each gene. Each bar represents the mean plus S.E. and the asterisk (*) indicates a significant difference between the level of wild-type and TRDKO with (T3) or without T3 (Control) treatment (*P* < 0.05)

KEGG pathway and GO analyses revealed that surprisingly, cellular senescence was the most significantly enriched among the 92 DEGs specific to nonregenerative animals (Fig. 5B, Table S7A), suggesting that T3-induced cellular senescence may inhibit tail regeneration (also see Fig. 6 below). However, regulation of actin, nervous system development and multicellular organism development were specifically highly enriched among the 370 DEGs in regenerative animals (Fig. 5C, Table S7.B). Consistent with the above results in Fig. 4B-C, cell cycle was significantly enriched among the 607 DEGs common to both regenerative and nonregenerative animals. In support of this, RNA-seq data of relative FPKM values and RT-qPCR analyses showed that the expression of 2 cell cycle genes, *cdca8* and *cdk2*, were upregulated at 24 h after amputation and exhibited the similar regulation pattern in the regenerative and nonregenerative animals during the initial period of regeneration (Fig. S4). These results indicate TR-mediated, T3-induced the regulated DEGs involved in regulating the cellular senescence may be responsible for the inhibition of tail regeneration in wild-type animals during at the climax of metamorphosis.

### TR is required for T3-inhibition of tail regeneration in premetamorphic tadpoles

The RNA-seq analyses above suggest that TRDKO regulates gene expression program during tail resorption to affect tail regeneration capacity. To test this, we studied the effect of T3-treatment on tail regeneration in premetamorphic WT and TRDKO tadpoles at stage 56. The results showed that three days of T3 treatment significantly inhibited tail regeneration in WT (Fig. 6A, C) but not TRDKO tadpoles (Fig. 6B, D). Moreover, 3-day T3 treatment induced the expression of cellular senescence marker genes p15 and p21 strongly, and to a lesser extent p53, in the regenerated portion of tail of WT tadpoles (Fig. 6E). In contrast, this T3 regulation was abolished for p15 and greatly reduced for p21 in the regenerated portion of tail of TRDKO animals compared to WT tadpoles (Fig. 6F), although p53 had little regulation by T3 in both TRDKO and WT tadpoles. Importantly, T3-treatment at stage 56 does not inducible any measurable tail resorption in either WT or TRDKO tadpoles. These results demonstrate that TR-mediated, T3-induced gene expression program inhibits tail regeneration in premetamorphic tadpoles. Thus, the inhibition of tail regeneration by T3-TR signaling is a direct effect, not an indirect consequence of tail resorption, likely via the activation of cellular senescence to change the permissive environment important for the initial period of tail regeneration in *Xenopus tropicalis*.

## Discussion

Regeneration is a multi-step process involving wound healing, blastema formation and the subsequent tissue patterning and outgrowth [5, 15, 36]. We have previously reported that the TR-mediated, T3-induced gene regulation program is responsible for the loss of tail regeneration capacity, particularly preventing patterning and outgrowth [5]. Of note, the initial period of regeneration and early structure of the regenerated tissue determine the extent of the functional recovery in the later phase of tissue regrowth [37]. However, the gene expression program underlying this early period remains poorly defined. Here, we applied transcriptomics approaches to investigate gene regulation underlying the initiation phase of tail regeneration (i.e., wound healing and blastema formation) and its regulation by T3 via TRs. Our findings suggest that TR-mediated, T3-induced gene regulation alters the permissive environment, e.g., induced cellular senescence, dysregulated inflammatory response and ECM remodeling during the initial period of regeneration, to affect the patterning/outgrowth period of the regeneration process.

Consistent with the previous studies in various species [33, 37–40], we observed the involvement of inflammatory response, metabolic process, collagen deposition, cell migration and cell apoptosis during initial wound healing period post tail amputation. Notably, proinflammation in the initial stages of the regenerative process activates the immune system to protect against infections and stimulates the removal of tissue debris [37]. For example, zebrafish neutrophils rapidly accumulate at wounds through various injury cues and engulf small dead cell debris, much like their mammalian counterparts [41]. Here, we also found that GO terms for the neutrophil migration was most significantly enriched during wound healing. Epithelial cell migration is required for forming the specialized wound epidermis that directs growth and patterning of the appendage such as the mobilization of fin-resident regeneration-organizing cells [33, 42, 43]. Consistently, we found that GO term for epithelial cell migration was also highly enriched (Fig. 2B). Thus, there is an evolutionally conserved gene regulation program during wound healing.

Apoptosis plays an indispensable role in wound healing, which allows for the elimination of cells without tissue damage or inflammatory response and thus helps the tissue to progress to the next phase of healing [31, 38, 44]. The dysregulation of apoptosis can lead to pathologic forms of wound healing such as excessive scarring. Interestingly, our previous study [5] and results in this study have showed that TR-mediated, T3-induced gene regulation affects apoptosis early during regeneration. Though both regenerative and nonregenerative animals can form wound epidermis, we observed that the 150 DEGs uniquely expressed in regenerative animals were highly enriched in genes for apoptosis (Fig. 1 and data not shown). Apart from *caspase9*, *bax* and *fas* gene, we found four other genes (i.e., *Imnb2*, *traf1*, *nfkbia*, *prf1*, *ctsw*) important for apoptosis were inhibited in the nonregenerative animals (Fig. S5). Thus, although both regenerative and nonregenerative animals can successfully form wound epidermis, T3/TRs-regulated genes may decrease the apoptotic response during wound healing in nonregenerative animals. This lower level of apoptosis may lead to incomplete or untimely elimination of the unwanted cells after amputation, thus limiting functional cell populations, e.g., progenitor cell, epithelial cell, during the early regeneration period and adversely affecting subsequent cell proliferation and tissue patterning [44].

In addition, we observed several miRNAs, such as mir-222, mir-133a and mir-29a (Table S2), were regulated during wound healing, consistent with earlier report of miRNAs involvement in tissue remodeling and wound healing [45, 46].In particular, mir-29a and mir-222 are implicated in wound repair during wound healing, and the upregulation of mir-29a decreases scar formation by inhibiting TGF-b2/smad3 [47, 48]. Our finding that these two genes are also upregulated during *Xenopus* tail regeneration suggests their conserved role during wound healing.

Blastema formation serves as the bridge between the specialized wound epidermis and the outgrowth phase in appendage regeneration, which determines the eventual outcomes of tissue/organ regeneration and functional recovery [4, 34, 49, 50]. Interestingly, the genes involved in blastema formation in nonregenerative tails were mostly altered by the action of T3 via TRs during tissue regeneration. Functional enrichment of the DEGs identified cellular senescence, cytokine-cytokine receptor interaction and ECM-receptor interaction, suggesting that these dysregulated genes and biological processes are responsible for the loss of regeneration.

The balance of pro-inflammation and anti-inflammation has been implicated as a critical step in successful regeneration [51, 52]. Previous studies have demonstrated that prolonged inflammation leads to deficient wound closure, producing a scar that can finish with pathological fibrosis and commonly present in nonregenerative organs [53–56]. Consistently, the inflammation pathway and genes (i.e., *mmp13*, *il17c*) were observed persistently upregulated in the blastema formation period in nonregenerative animals while downregulated in regenerative animals (Fig. 4). Thus, T3 signaling may affect inflammatory response to influence the normal regenerative process [56].

In addition, ECM remodeling is essential for blastema formation [57–59]. Consistently, we found that ECM remodeling pathway and genes were upregulated in the blastema formation period in regenerative animals while downregulated in nonregenerative animals (Fig. 4, and Fig. S3), suggesting that the TR-mediated differential T3 signaling may cause dysregulation of ECM remodeling to affect the outcome of regeneration [7, 60]. Additionally, single-cell RNA-Seq and spatial transcriptomics analysis of *Xenopus* tail during regeneration revealed a new cell type (RIC, regeneration initiating cells) that is critical for the modification of the surrounding ECM to allow for migration of other cell types such as regeneration organizing cells (ROC) to direct regeneration [33, 37]. Here, we also found one of the RICs marker genes, *fibin*, was upregulated in the regenerative tail but downregulated in the nonregenerative tail (Fig. 4D), suggesting that T3 signaling may affect the gene expression of RICs to influence the ECM remodeling. Thus, this deprivation of ECM remodeling and inflammatory response gene/pathway expression by T3, via TR, may lead to regenerative failure in nonregenerative animals [7, 61]. Taken together, these findings indicate that TR-mediated, T3-induced genes alter the permissive environment, and eventually changing the tail regenerative outcomes in *Xenopus tropicalis*.

## Conclusions

In summary, by using wild-type and TR-double knockout animals at different metamorphic stages, we showed here that TR-mediated, T3-induced transcriptional regulation programs alter the tail regenerative permissive environment during the initial period of tail regeneration, and further impact the subsequent outgrowth. Specifically, T3 signaling via TRs inhibits the expression of ECM-related genes while promoting the expression of inflammation-related genes during the blastema formation period. Interestingly, our findings indicate that amputation-induced changes in DNA replication-related pathways can occur during this nonregenerative period. Future studies, particularly on the regenerative microenvironment that may depend on cytokine-cytokine receptor interaction and ECM-receptor interaction, should provide novel insight into the developmental regulation of regenerative capacity. Furthermore, understanding how TR regulates the biological processes and genes underlying the initial period of tail regeneration in the *Xenoups* tail may lead to the identification of key pathways or candidate genes for the potential avenues to retain generative capacity in adult vertebrate organs.

## Supplementary Information

Supplementary Material 1.

Supplementary Material 2.

## Data Availability

Supplementary data to this article can be found online. RNA sequencing data were deposited in the NCBI GEO database with accession number GSE225045.

## Abbreviations

T3: Thyroid hormone
TRDKO: TRα and TRβ knockout
WT: Wild type
hap: Hours post amputation
RT-qPCR: Quantitative reverse-transcription PCR
GO: Gene Ontology
KEGG: Kyoto encyclopedia of genes and genomes
FPKM: Fragments Per Kilobase of the transcript per Million fragments mapped
DEGs: Differentially expressed genes
ECM: Extracellular matrix
mmps: Matrix metalloproteinases
TGF: Transforming growth factor
IL: Interleukin
*col4a5*: Collagen Type IV Alpha 5 Chain
*fibin*: Fin Bud Initiation Factor Homolog
*bax*: Bcl2-associated X protein
*traf1*: TNF receptor associated factor 1
*Imnb2*: Lamin B2
*prf1*: Perforin 1
*ctsw*: Cathepsin W
cdk1/2: Cyclin-dependent kinase 1/2
cdca8: Cell division cycle associated 8
miRNA: MicroRNA
ROC: Regeneration organizing cells
RIC: Regeneration initiating cells
