# Supplementary material for: Thyroid hormone receptor- and stage-dependent transcriptome changes affect the initial period of Xenopus tropicalis tail regeneration
Source: BMC Genomics. 2024 Dec 31;25:1260. doi: 10.1186/s12864-024-11175-4 (PMC11686881; doi:10.1186/s12864-024-11175-4)
Supplement: Supplementary file 1 — Supplementary Material 1. [file 12864_2024_11175_MOESM1_ESM.pdf]

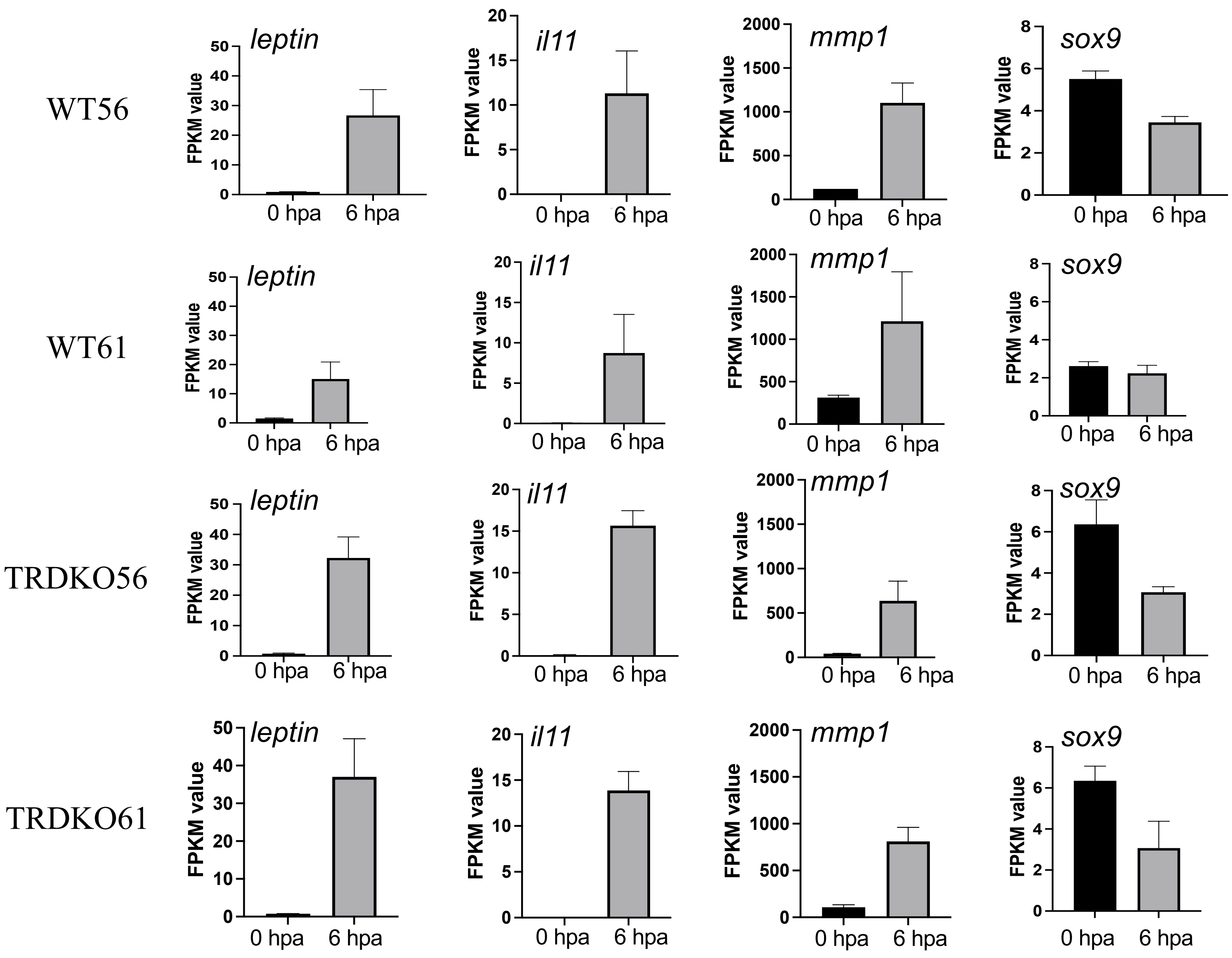

Fig. S1. The expression of some genes known to be regulated during wound healing. Note that all four genes (leptin, il11, mmp1,sox9) have similar expression patterns in the regenerative and nonregenerative animals.

A

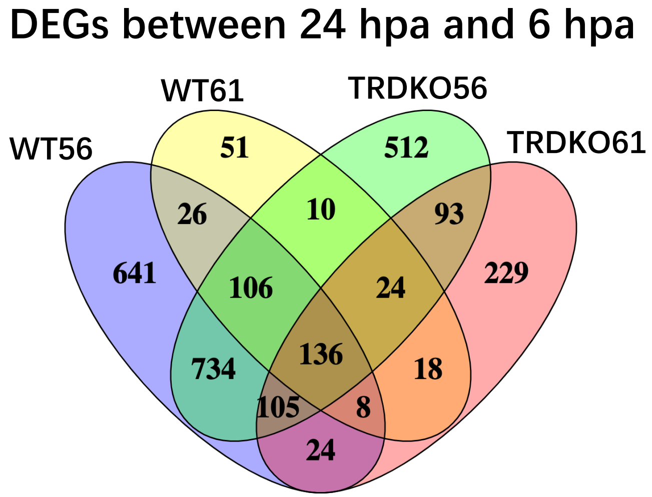

Fig. S2. The common and unique biological processes enriched among DEGs during blastema formation in the regenerative and nonregenerative animals.

(A) Venn diagrams comparison of the DEGs in the 4 groups of animals.

(B) The top 5 KEGG pathways and GO terms enriched among the 136 DEGs common to all 4 groups. Note that DNA replication was highly enriched among the common regulated genes.

(C) The top 5 KEGG pathways and GO terms enriched among the 105 DEGs common to the regenerative animals. Note that Cytokine-cytokine receptor interaction and ECM-receptor interaction were highly enriched in regenerative animals.

B

#	KEGG pathways	GO terms
1	DNA replication	Cell cycle
2	Pyrimidine metabolism	Mitotic cell cycle
3	Cell cycle	Cell cycle process
4	Mismatch repair	Mitotic cell cycle process
5	Drug metabolism - other enzymes	Chromosome organization

C

#	KEGG pathways	GO terms
1	Arginine and proline metabolism	DNA-templated DNA replication
2	Cell cycle	DNA replication
3	Fanconi anemia pathway	Mitotic cell cycle
4	Cytokine-cytokine receptor interaction	DNA unwinding involved in DNA replication
5	ECM-receptor interaction	Positive regulation of DNA primase activity

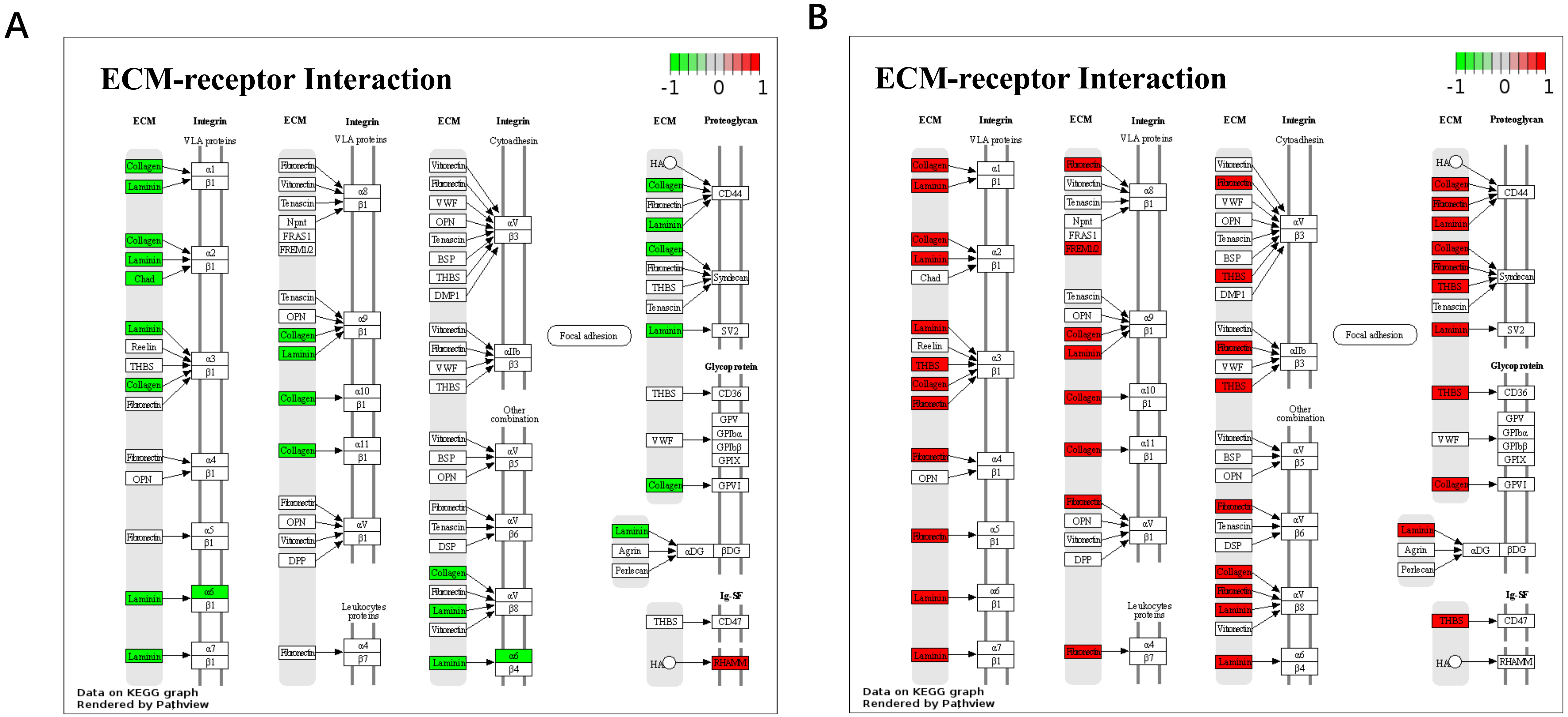

Fig. S3. The regulation of genes in the ECM-receptor interaction pathway in nonregenerative (A) and regenerative animals (B) during blastema formation (see Fig. 4). Note that all DEGs (shown in red or green) except one (HA) were downregulated in the nonregenerative tail whereas all DEGs were upregulated in the regenerative tail.

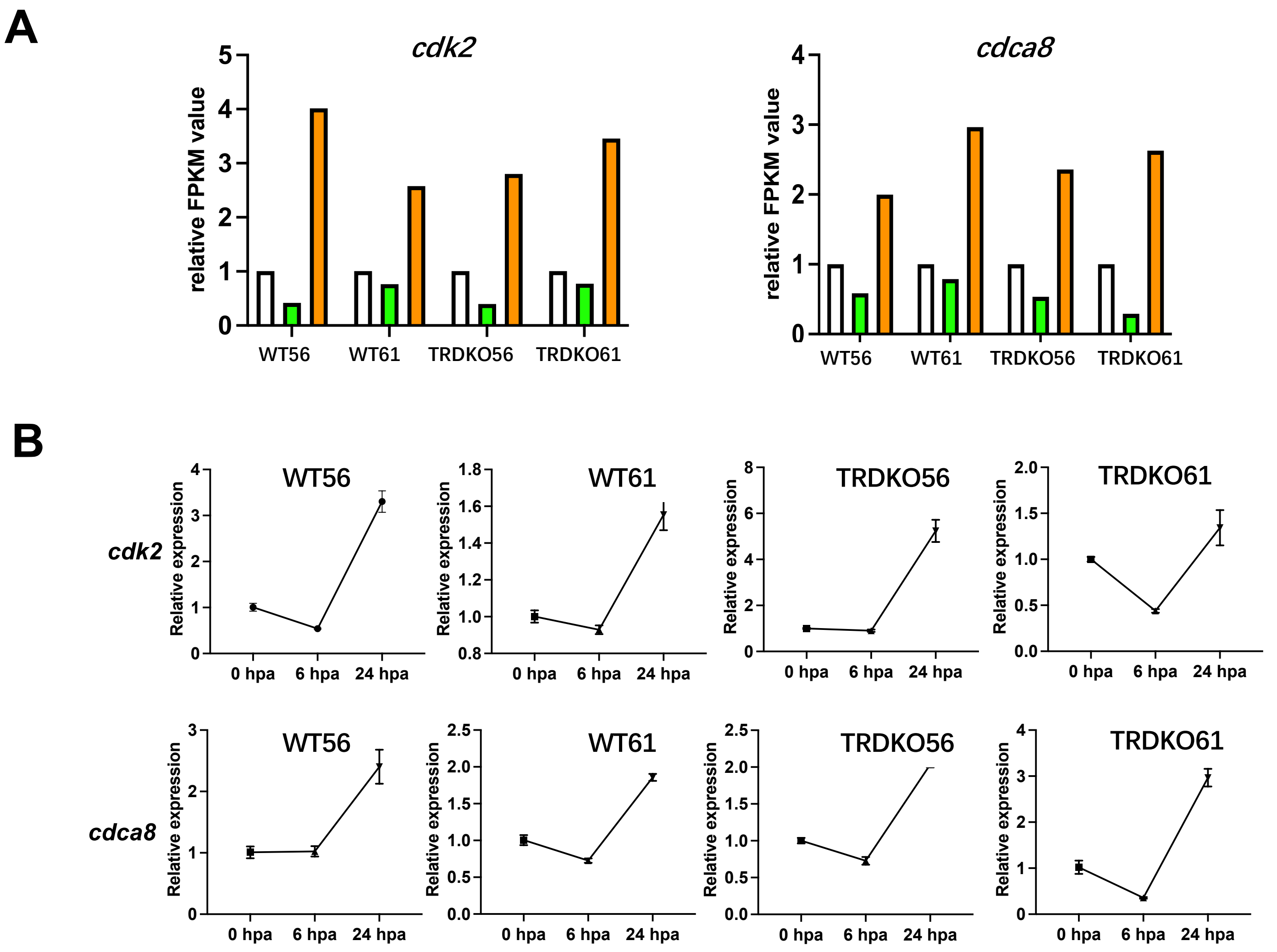

Fig. S4. Cell cycle genes are upregulated at 24 hpa (blastema period) in all 4 groups after tail amputation.

(A) FPKM value showed the expression of 2 cell cycle genes, *cdk2* and *cdca8*.

(B) The regulation of *cdk2* and *cdca8* was confirmed by RT-qPCR, normalized to that of *rpl8*. Note that both genes were upregulated at 24 hpa and had a similar expression pattern as obtained from the RNA-seq data in (A).

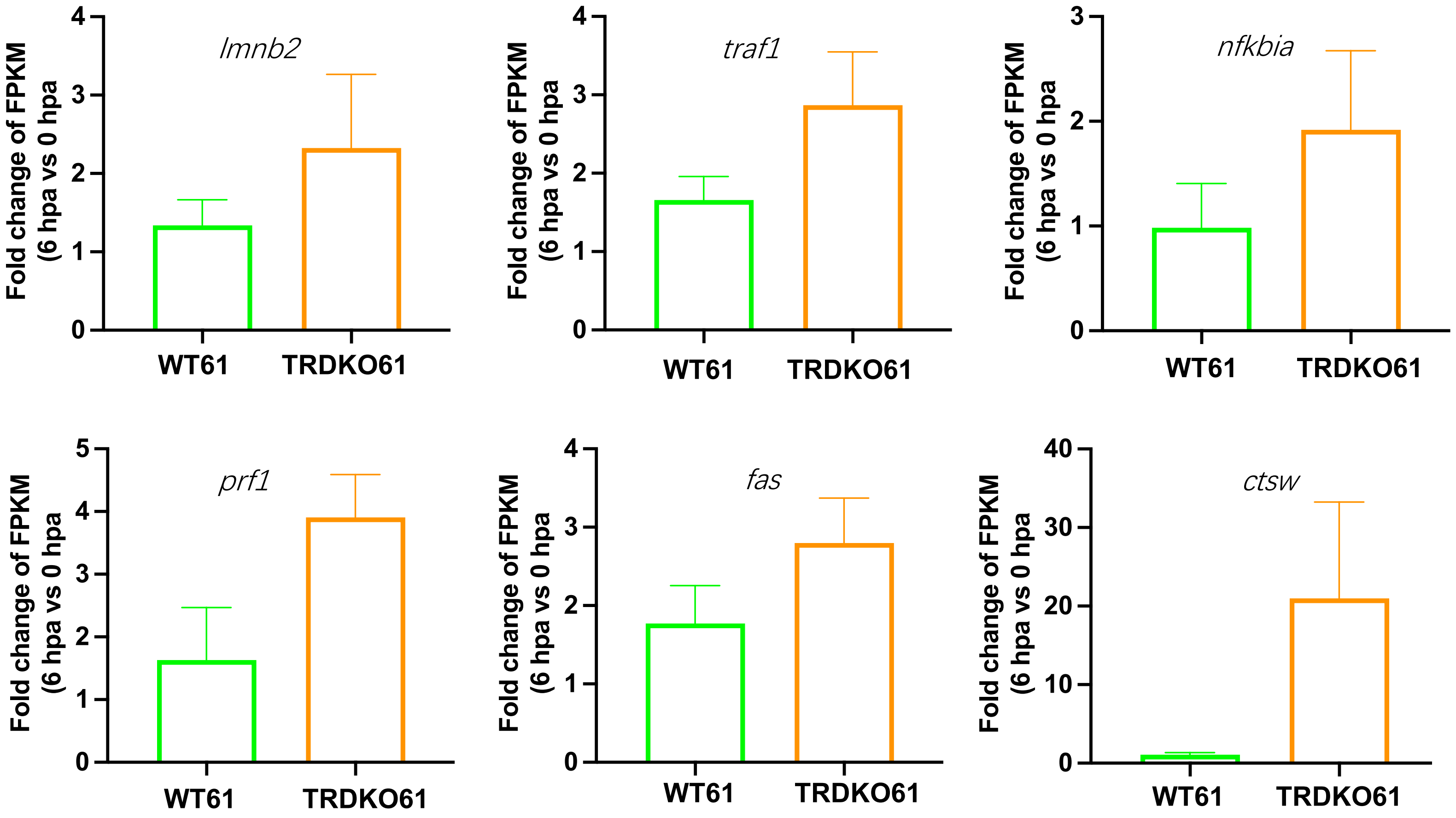

Fig. S5. TRDKO increases the induction of the apoptotic genes during the wound healing period at stage 61. The expression of six genes involved in the apoptosis pathway, *lmnb2*, *traf1*, *nfkbia*, *prf1, fas and ctsw*, was shown as the ratio of the FPKM value at 6 hpa to that at 0 hpa.
